# Molecular prevalence of *Toxoplasma gondii* and *Trypanosoma evansi* in recently calved female cattle from Phayao, Thailand

**DOI:** 10.14202/vetworld.2024.756-762

**Published:** 2024-04-07

**Authors:** Khuruwan Klinbumrung, Khanuengnij Prakhammin, Ornampai Japa

**Affiliations:** 1 Scientific Instrument and Product Standard Quality Inspection Center, University of Phayao, Phayao, Thailand; 2 Department of Applied Statistics, Rajamangala University of Technology Isan, Khon Kaen Campus, Khon Kaen, Thailand; 3 Division of Microbiology and Parasitology, School of Medical Sciences, University of Phayao, Phayao, Thailand

**Keywords:** bovine placenta, *Toxoplasma gondii*, transplacental transmission, transplacental-transmitted protozoan, *Trypanosoma evansi*

## Abstract

**Background and Aim:**

*Toxoplasma gondii* and *Trypanosoma evansi*, the zoonotic protozoa responsible for toxoplasmosis and trypanosomiasis, are significant threats to the productivity and financial stability of livestock farming. *T. gondii* can be transmitted horizontally through ingestion of fecal oocysts and *T. evansi* through arthropod vectors. In addition, both species can be transmitted from mother to fetus through the placenta. This study aimed to assess the molecular prevalence of *T. gondii* and *T. evansi* transplacental-transmitted protozoans and to identify the epidemiological risk factors in recently calved female cattle across Phayao, Thailand.

**Materials and Methods:**

We collected 106 bovine placentas from beef and dairy cow full-term pregnancies in Phayao, Thailand. *T. gondii* and *T. evansi* DNA were detected using targeted B1 gene and expression site-associated gene (ESAG) species-specific polymerase chain reaction (PCR), respectively.

**Results:**

Forty-three placentas were positive for *T. gondii* B1 PCR, whereas only one was positive for *T. evansi* ESAG PCR, resulting in an overall prevalence of transplacental-transmitted protozoan infection of 41.5% (44/106). The prevalence of *T. gondii* and *T. evansi* was 40.6% (43/106) and 0.9% (1/106), respectively. No significant correlation was found between *T. gondii* infection and various risk factors, including locality, age, and cattle type.

**Conclusion:**

The prevalence of transplacental-transmitted protozoan *T. gondii* infection was high among female cattle in Phayao, Thailand, whereas the prevalence of *T. evansi* infection was notably lower. Although the conventional modes of transmission differ between these two parasites, the transplacental transmission of *T. evansi* and especially *T. gondii* may play a crucial role in the persistence of these protozoan species in this area.

## Introduction

*Toxoplasma gondii* and *Trypanosoma evansi* are protozoa of significant zoonotic importance, impacting both animal and human health globally [1–3]. As causative agents of toxoplasmosis and trypanosomiasis in animals, they pose substantial threats to livestock farming, affecting productivity and farmers’ financial stability [4–6].

*T. gondii* is an intracellular protozoan with a broad host range that encompasses warm-blooded animals, including humans [7, 8]. It is one of the most successful and widely distributed parasitic protozoans [8]. During the lifecycle of *T. gondii*, felids, particularly domestic cats, are definitive hosts and play a crucial role in spreading the disease by excreting a substantial number of *T. gondii* oocysts in feces [4, 8]. The clinical manifestations of toxoplasmosis can vary among animal hosts, including fever, lymphadenopathy, eye inflammation, and central nervous system disruption [9]. Although infections in immunocompetent animals are generally asymptomatic, the parasite may cause severe consequences [10]. For example, toxoplasmosis can result in abortion for pregnant women or animals at their first exposure [11] and life-threatening for animals with compromised immune system [10, 12, 13].

*T. evansi*, a hemoflagellate protozoan, is the causative agent of trypanosomiasis, commonly known as surra. This disease primarily affects domestic and wild mammals such as dogs, cats, cattle, buffaloes, horses, and camels [14]. The clinical manifestations of surra include fever, anemia, weakness, emaciation, and central nervous system disorders [15]. In severe cases, the infection may be fatal [14].

The presence of *T. gondii* and *T. evansi* can lead to serious health problems in livestock production. Bovine trypanosomiasis can present a spectrum of severity, ranging from mild to severe symptoms such as fever, anemia, weakness, weight loss, and lymph node enlargement. Severe cases may also show neurological manifestations [14, 16]. Conversely, clinical signs of toxoplasmosis in cattle are generally uncommon and often subclinical under normal conditions [4, 17]. However, during acute infection, affected cattle may exhibit mild symptoms such as fever, lethargy, and dyspnea [18]. *T. gondii* and *T. evansi* infections in pregnant cows can lead to reproductive complications, such as abortion, stillbirth, or the birth of weak or debilitated offspring [19, 20]. Although congenital cases of both diseases are rare in cattle, there is still a risk of infection resulting in reproductive failure. These infectious diseases have significant economic consequences for livestock production, resulting in significant production losses and morbidity [4, 16]. In addition, there is a risk of transmission to other animals and humans in affected areas [4], which highlights the need for robust control and prevention measures to protect animal well-being and public health.

The protozoan *T. gondii* is horizontally transmitted to cattle and other animals through the ingestion of shed oocysts by a cat or feline host [21, 22]. In contrast, *T. evansi* transmission in cattle and other large animals is mechanically transmitted by blood-sucking arthropods and biting insects, such as tabanid flies [23]. During pregnancy, immunological alterations in cattle may jeopardize their immune defenses against *T. gondii* and *T. evansi*, increasing the likelihood of vertical transmission to the fetus and leading to adverse reproductive outcomes. Vertical transmission can also occur through transplacental transmission of active parasites from infected mothers to the fetus [8, 14].

In our previous study, we presented preliminary findings indicating a notable prevalence of transplacental-transmitted protozoan *Neospora caninum* in female beef cattle in Phayao, Thailand [24]. Other transplacental zoonotic protozoal infections remain unconfirmed, including *T. gondi*i and *T. evans*i. Therefore, the aim of this study was to assess the molecular prevalence of transplacental-transmitted protozoans *T. gondii* and *T. evansi* and to identify epidemiological risk factors in female cattle who have recently calved.

## Materials and Methods

### Ethical approval

The University of Phayao Animal Ethics Committee granted ethical approval for the utilization of animals in this study, with the protocol number UP-AE59-02-04-0001.

### Study period and location

This study was conducted from January to December 2016 throughout the year. Bovine placenta specimens were obtained from cattle reared in nine districts of Phayao province: Muang, Mae Chai, Dok Kamtai, Chiang Kham, Chiang Muan, Pong, Phu Sang, Phu Kamyao, and Chun. All laboratory examinations were performed at the School of Medical Sciences and the Scientific Instrument and Product Standard Quality Inspection Center, University of Phayao, Phayao, Thailand.

### Animals and specimen collection

One hundred and six bovine placental specimens were collected from cows within 6 h of full-term pregnancy and calving. Cotyledons from each placenta were carefully isolated and subsequently preserved at –20°C until molecular analysis. Beef and dairy cows aged between 2 and 8 years were studied, all of which were reared by small-scale farmers in nine districts of Phayao province (with <30 cows). Detailed records of the age and breed of each animal were recorded.

### Extraction of placental DNA

Bovine placental DNA was extracted and purified using a QIAamp DNA preparation kit (Qiagen, Germany) according to the manufacturer’s instructions. In brief, 100 mg of cotyledon tissue was homogenized in 180 μL of ATL buffer and 20 μL of a mixture of proteinase K and RNase A, followed by incubation at 56°C for 1 h. After incubation, 200 μL of AL buffer was added and thoroughly mixed by vortexing for 15 s before adding 200 μL of absolute ethanol. The mixture was then incubated at room temperature (25°C) for 10 min, loaded into the QIAamp MinElute (Qiagen) column, and centrifuged at 12,000× *g* for 1 min. The spin column was washed with 500 μL of AW1 buffer and centrifuged for 1 min after centrifugation. The washing process was repeated with 500 μL of AW2 buffer. Genomic DNA was eluted using 100 μL of AE buffer and stored at –20°C until further use.

### *T. gondii* and *T. evansi* DNA polymerase chain reaction (PCR) screening

PCR was performed in a reaction volume of 50 μL, using QIAGEN Taq DNA Polymerase (Qiagen, Germany). In each PCR reaction, 200 μM of each dNTP, 1.5 mM of MgCl_2_, 10 pmol of each species-specific primer [25, 26], and 1.25 U of Taq DNA polymerase were added to coral PCR buffer with a pH of 8.7. In addition, 2 μL of the extracted DNA template was added to the reaction mixture. Table-1 [25, 26] summarizes the primer sequences and the expected PCR amplicons.

**Table-1:** The sequence of PCR primers and the expected PCR products for the molecular detection of *T. gondii* and *T. evansi* in bovine placenta.

Gene	Primer	Sequences (5’-3’)	PCR product (bp)	Reference
*T. gondii* (B1)	B22 B23	AACGGGCGAGTAGCACCTGAGGAGA TGGGTCTACGTCGATGGCATGACAAC	115	[25]
*T. evansi* (ESAG)	ESAGF ESAGR	ACATTCCAGCAGGAGTTGGAG CACGTGAATCCTCAATTTTGT	238	[26]

*T. gondii*=*Toxoplasma gondii*, *T. evansi*=*Trypanosoma evansi*, PCR=Polymerase chain reaction, ESAG=Expression site-associated gene

For *T. gondii* DNA detection, the following PCR conditions were used: 5 min at 94°C for denaturation, 40 cycles of 94°C for 30 s, 60°C for 30 s, 72°C for 1 min, and 5 min of a final extension step at 72°C. For *T. evansi* PCR, the amplification protocol was similar to that of B1 PCR, except that the annealing temperature was 55°C. The amplification reaction was performed using a GeneAmp PCR System 9700 (Applied Biosystems, USA).

DNA from *T. gondii* and *T. evansi* was used as a positive control, whereas that from *N. caninum* reference isolates (Nc-1) was used as the negative control.

PCR-amplified products were analyzed through electrophoresis on a 1.5% agarose gel in tris, acetate, and EDTA buffer. Agarose gel was pre-stained with Nancy-520 (Sigma-Aldrich, UK). PCR fragments were randomly selected for sequencing among the positive samples to validate the precision and specificity of the molecular detection method. The PCR products were sequenced in the forward and reverse directions using the respective primers. DNA sequencing was performed using Macrogen Sequencing (Korea).

### Sequence analyses

Sequences of positive PCR from *T. gondii* and *T. evansi* were carefully checked and manually assembled. The obtained nucleotide sequence was initially analyzed using the standard nucleotide Basic Local Alignment Search Tool, accessible through the National Center for Biotechnology Information database (http://blast.ncbi.nlm.nih.gov/).

### Accession numbers for nucleotide sequence

The *T. gondii* B1 sequences obtained in this study were registered in the GenBank database with the following accession numbers: OR853742-OR853745. The nucleotide sequence of *T. evansi* expression site-associated gene used in this study was deposited in the GenBank database under the accession number OR853746.

### Statistical analysis

Descriptive statistics were used to determine the prevalence of *T. gondii* and *T. evansi* infection in each location, age group, and cattle type. The prevalence of infection was determined according to the ratio of positive PCR results to the total number of placentas examined. Differences in prevalence among distinct locations, age groups, and cattle types were analyzed using the Chi-square test. Statistical significance is indicated by p < 0.05.

## Results

### Cattle samples

A total of 106 female cattle placentas were collected shortly after calving for the examination of *T. gondii* and *T. evansi* DNA. The distribution of samples from each district was as follows: Mae Chai (38), Dok Kamtai (19), Chiang Kham (13), Muang (12), Chiang Muan (12), Pong (6), Phu Sang (4), Phu Kamyao (1), and Chun (1). The majority of placentas (98) were obtained from beef cattle, while eight placentas originated from dairy cattle. The mean age was 4.5 years (range 2–8 years). The majority of female cattle were <5 years old.

### Molecular detection of *T. gondii* and *T. evansi* transmitted placentally

**Figure-1:**
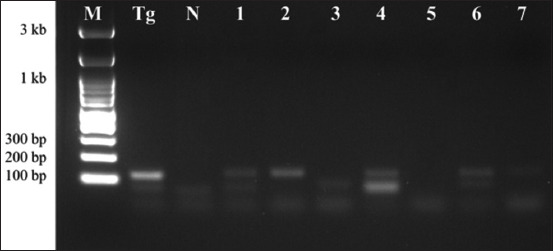
Representative polymerase chain reaction detection of *Toxoplasma gondii* in bovine placentas from cattle in Phayao, Thailand. Lane M: 100 bp DNA ladder; lane Tg: Positive control (reference strain *T. gondii*); lane N: Negative control (*N. caninum*); lanes 1–2, 4, and 6–7: Positive *T. gondii* samples; lanes 3 and 5: Negative samples.

**Figure-2:**
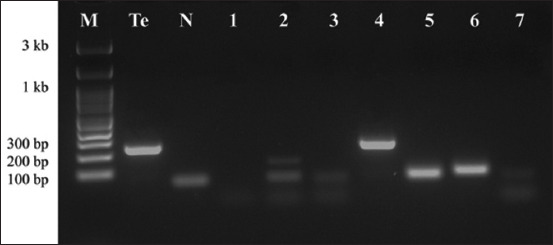
Representative polymerase chain reaction detection of *Trypanosoma evansi* in bovine placenta from cattle in Phayao, Thailand. Lane M: 100 bp DNA ladder; lane Te: Positive control (reference strain *T. evansi*); lane N: Negative control (*N. caninum*); lane 4: Positive *T. evansi* sample; lanes 1–3 and 5–7: Negative samples.

*T. gondii* and *T. evansi* DNA were examined in the placentas. Of the 106 specimens examined, 43 were positive for *T. gondii* B1 PCR, whereas only one was positive for *T. evansi*. *T. gondii* PCR analysis successfully amplified a 115-bp fragment of the B1 gene (Figure-1), whereas *T. evansi* PCR screening yielded a 238-bp amplicon from a single placenta of a 4-year-old beef cow from the Mae Chai district (Figure-2). Notably, there were no mixed infections in the samples.

### Sequence analyses

Positive samples were randomly selected for confirmation by sequencing. The nucleotide sequences obtained by *T. gondii* PCR exhibited a perfect match (98.26%–100% similarity) to the *T. gondii* B1 sequences deposited in GenBank (LN714499.1, MZ027344.1, AF179871.1, and XM_002370240.2). Sequencing results for positive *T. evansi* showed 98.73% similarity with GenBank sequences (KR858289.1, KR858291.1, KR858293.1, and KR858301.1).

### Molecular prevalence and risk factor analyses

Only one placental sample from Mae Chai district tested positive for *T. evansi* by PCR, resulting in a prevalence of 0.9%. Positive placenta belonged to a 4-year-old beef cow.

On the other hand, 43 placentas tested positive for *T. gondii* DNA, indicating a prevalence of 40.6%. *T. gondii*-positive samples were found in placentas collected from all districts, with a prevalence ranging from 31.6% to 100.0%. The highest prevalence of *T. gondii* was observed in placentas from Phu Kamyao (100.0%), Chun (100.0%), Pong (66.7%), Muang (63.6%), and Phu Sang (50.0%), while the lowest prevalence was noted in cattle from Dok Khamtai district (31.6%). There were no significant differences in the proportions of positive cattle among different districts (p > 0.05).

The highest infection rate was observed in cattle aged ≥5 years (50.0%), followed by those aged <5 years (42.2%) and of unknown age (24.0%) among various age groups. With regard to cattle type, three positive samples for *T. gondii* PCR were found among the eight dairy cows (37.5%), whereas the remaining 40 positive samples were obtained from beef cattle (40.8%, 40/98). Statistical analysis revealed no significant correlation between cattle age and cattle type with *T. gondii* infection (p > 0.05). Table-2 presents the molecular prevalence of transplacental-transmitted protozoan *T. gondii* in naturally infected female cattle, categorized by each parameter.

**Table-2:** Molecular prevalence of *T. gondii* infection in naturally infected female cattle in Phayao according to location, age, and type of cattle.

Variables/Factors	No. of animals	No. of *T. gondii* infected animals (prevalence %)	Chi-square	p-value
Locality			5.1604	0.7403
Muang	12	7 (58.3)		
Mae Chai	38	12 (31.6)		
Dok Kamtai	19	6 (31.6)		
Chiang Kham	13	6 (46.2)		
Chiang Muan	12	4 (33.3)		
Pong	6	4 (66.7)		
Phu Sang	4	2 (50.0)		
Phu Kamyao	1	1 (100.0)		
Chun	1	1 (100.0)		
Age			2.5115	0.2849
<5 years	45	19 (42.2)		
≥5 years	36	18 (50.0)		
Unknown	25	6 (24.0)		
Cattle type			0.0201	0.8874
Beef	98	40 (40.8)		
Diary	8	3 (37.5)		
Total	106	43 (40.6)		

*T. gondii*=*Toxoplasma gondii*

## Discussion

This study assessed the prevalence of transplacental-transmitted *T. gondii* and *T. evansi* protozoans in cattle from Phayao, northern Thailand, by screening placental specimens for protozoan genetic material. Detection of parasite DNA in the placenta suggested migration of the protozoan through the maternal bloodstream, potentially causing fetal infection. A positive result would indicate an active infection in the pregnant cow, implying that the protozoan is likely to pass through the placenta to the developing fetus. Our results revealed that 40.6% of bovine placentas tested positive for *T. gondii* DNA. In comparison to *T. gondii*, only 0.9% were positive for *T. evansi*, indicating that *T. gondii* is likely a common and more prevalent parasite associated with vertical transmission among cattle in Phayao. The distribution of *T. gondii* was consistently observed across all Phayao districts, although these differences did not reach statistical significance. On the other hand, *T. evansi* infection was identified in only one specimen collected from Mae Chai district, corresponding to a prevalence of 0.9%.

Notably, the overall prevalence of *T. gondii* in our study was higher than that reported in other regions of Thailand [27–29]. For example, recent investigations in the western regions reported a *T. gondii* infection rate of 15.2% in beef cattle [27], whereas serological examinations in the northern provinces documented *T. gondii* infection rates of 9.4%–17.0% in dairy cows [29]. The high prevalence of *T. gondii* in pregnant cows underscores the importance of understanding and addressing the impact of *T. gondii* on animal health and human health.

*T. gondii* and *N. caninum* present significant concerns for the livestock industry, resulting in substantial economic losses in ruminants [8]. In this study, we investigated placental infections of both *T. gondii* and *T. evansi*. We used bovine placentas collected from the same area as in our previous study, which determined the prevalence of *N*. caninum transplacental-transmitted protozoan [24]. If we combine the results of this study with those earlier findings, a mixed infection of *T. gondii* and *N. caninum* in beef cattle was identified in 16.0% of cows, a rate lower than 27.2% reported by Udonsom *et al*. [27] in their study in three provinces of western Thailand. This difference in prevalence can be attributed to differences in assessment methodologies and specific animal populations examined. Importantly, there was no evidence of a mixed infection of *T. evansi* with either *T. gondii* or *N. caninum* in this study.

Cattle are generally considered to be more resistant to *T. gondii* infection, and the occurrence of *T. gondii*-induced abortion in cattle is relatively rare [4, 30, 31]. However, the severity of the disease depends on a number of factors involving both the host and the parasite. Host factors, such as host species and individual immune systems, may affect clinical manifestations and susceptibility to infection, particularly during pregnancy and lactation or in undernourished animals. Pathogen-related factors, such as infection dosage, *T. gondii* genotype, and route of infection, also contribute to the overall outcome [4, 31].

One significant factor is the genotype of *T. gondi*i, which is generally classified into three genotypes (I-III), with genotype I being the most virulent [32]. In humans and animals, this genotype is associated with more severe clinical manifestations, including tissue damage, organ failure, and even death [33, 34]. It should be noted that a virulent genotype in a particular area may increase the risk of serious infections and may affect the overall epidemiology of toxoplasmosis in that region. Suwancharoen *et al*. [35] and Chemoh *et al*. [36] recently presented evidence of *T. gondii* genotype I in definitive cat hosts in northern and southern Thailand, respectively. These findings highlight the potential risk of *T. gondii* genotype I exposure to cattle and humans. Therefore, further investigation into the circulation and dynamics of *T. gondii* genotype among hosts, including cattle, is essential for devising effective management and control strategies.

Our preliminary findings on the prevalence of *T. evansi* infection in female cattle in Phayao showed a relatively low presence of the parasite in bovine placentas compared to seroprevalence rates reported in other regions of Thailand [16, 37–41]. *T. evansi* infection in cattle is predominantly characterized as a subclinical disease, and cattle are often considered reservoir hosts for infection by other local susceptible hosts [14]. Despite this prevailing characterization, *T. evansi*-induced acute trypanosomiasis in cattle has been documented in India [42] and Thailand [15]. The economic impact of *T. evansi* infection on herds, affecting both small- and large-scale farms, is evident and primarily manifested in reduced productivity, leading to a subsequent decline in farmers’ incomes. Pholpark *et al*. [43] found a correlation between *T. evansi* infection and significant decreases in milk production in dairy cows. In addition, cases of abortion linked to *T. evansi* have been documented [16, 44]. The low prevalence of *T. evansi* observed in this study still poses a risk, suggesting that transplacental transmission may contribute to the sustainability and persistence of this zoonotic parasite.

## Conclusion

This study revealed a high prevalence of *T. gondii* transplacental transmission, whereas the prevalence of *T. evansi* transplacental transmission was notably low in Phayao, northern Thailand. Although *T. gondii* and *T. evansi* are classically transmitted through different mechanisms, the transplacental transmission of *T. gondii* and *T. evansi* appears to play a significant role in sustaining and contributing to the persistence of these protozoan species in this area. The information presented here has significant implications for future research and intervention strategies of these important zoonotic protozoa, as they could harm livestock productivity and pose a significant health risk for both animals and humans.

## Authors’ Contributions

OJ: Conceived and designed the study. OJ and KK: Surveyed, collected placental samples, and performed the study. OJ and KK: Conducted molecular work and performed sequence analyses. OJ and KP: Analyzed the data. OJ, KP, and KK: Drafted and revised the manuscript. All authors have read, reviewed, and approved the final manuscript.
